# Hedgehog Signaling in Skeletal Development: Roles of Indian Hedgehog and the Mode of Its Action

**DOI:** 10.3390/ijms21186665

**Published:** 2020-09-11

**Authors:** Shinsuke Ohba

**Affiliations:** Department of Cell Biology, Institute of Biomedical Sciences, Nagasaki University, Nagasaki 852-8588, Japan; s-ohba@nagasaki-u.ac.jp; Tel.: +81-95-819-7630

**Keywords:** hedgehog, skeletal development, endochondral ossification

## Abstract

Hedgehog (Hh) signaling is highly conserved among species and plays indispensable roles in various developmental processes. There are three Hh members in mammals; one of them, Indian hedgehog (Ihh), is expressed in prehypertrophic and hypertrophic chondrocytes during endochondral ossification. Based on mouse genetic studies, three major functions of Ihh have been proposed: (1) Regulation of chondrocyte differentiation via a negative feedback loop formed together with parathyroid hormone-related protein (PTHrP), (2) promotion of chondrocyte proliferation, and (3) specification of bone-forming osteoblasts. Gli transcription factors mediate the major aspect of Hh signaling in this context. Gli3 has dominant roles in the growth plate chondrocytes, whereas Gli1, Gli2, and Gli3 collectively mediate biological functions of Hh signaling in osteoblast specification. Recent studies have also highlighted postnatal roles of the signaling in maintenance and repair of skeletal tissues.

## 1. Introduction

Hedgehog (Hh) signaling plays essential roles in various developmental process in vertebrates and insects, and is a highly conserved pathway among species. Hh was first identified as a segment polarity gene by extensive screening for genes responsible for the *Drosophila* body plan. The name Hedgehog refers to the disorganized bristles covering the *hh*-null embryos, which resemble hedgehog spines. Hh is secreted from a certain population in developing tissues, travels over the tissues, and regulates the activity of cells as a morphogen, which regulates the activity of recipient cells according to its concentration. The range that Hh diffuses over varies depending on the tissues: Up to 50 μm in the imaginal disc of *Drosophila* and 300 μm in the limb bud of vertebrates [1,2].

Mammals have three hedgehog genes: Sonic hedgehog (Shh), Indian hedgehog (Ihh), and Desert hedgehog (Dhh). Shh is involved in the L-R axis determination, the patterning of body segments, the neural tube, and limbs and morphogenesis of the hair, teeth, lungs, gut, and muscle. In particular, Shh expressed in the zone of polarizing activity in developing limbs and in the notochord and floor plate of the neural tube determines the patterning of digits and cell fates of neural progenitors, respectively. Dhh regulates spermatogenesis and the formation of the peripheral nerve sheaths. Ihh is implicated in angiogenesis and hematopoiesis as well as skeletal formation [1,2].

A large number of studies have revealed various and indispensable functions of Hh signaling for the development and maintenance of skeletal tissues, as several concise and comprehensive reviews recently overviewed [3,4,5]. This review particularly aims to organize and summarize the functions of Hh signaling and the mode of Hh action in skeletal development, based mainly on the findings of mouse genetic studies.

## 2. Two Ossification Processes in Mammals

Skeletal elements develop through two ossification processes in mammals: Intramembranous ossification and endochondral ossification. In intramembranous ossification, mesenchymal cells condense at the region where future bone is formed. The condensed mesenchyme directly differentiates into bone-forming osteoblasts. Intramembranous bones include the frontal bone, parietal bone, facial bone, part of the temporal bone, and part of the clavicle.

Endochondral ossification begins with the formation of the cartilage mold; the condensed mesenchyme initially differentiates into chondrocytes, which produce a variety of glycosaminoglycan and cartilage matrix proteins, including type II, type IX, and type XI collagens. Chondrocytes proliferate and maturate. Terminal differentiation of chondrocytes is characterized by its post-mitotic hypertrophy; the hypertrophic chondrocytes express type X collagen, matrix metalloproteinase 13 (MMP13), and vascular endothelial growth factor (VEGF) and induce calcification of their surrounding matrixes. Vascular invasion is facilitated by the aforementioned vascularization-promoting factors. Hypertrophic chondrocytes eventually undergo apoptosis. The cartilage matrixes are then absorbed by chondroclasts, which are recruited into the developing cartilage along with vascular invasion.

The fibrous layer that surrounds the developing cartilage is called the perichondrium, and it is known as a source of osteo-chondroprogenitors. Some cells in the perichondrium are specified into the osteoblast lineage. The perichondrial cell-derived osteoblasts form the bone collar, a predecessor of cortical bones. The specified population becomes bone-forming osteoblasts through several precursors. Upon their specification, perichondrial cells initially give rise to osteoblast precursors expressing runt-related transcription factor 2 (*Runx2*). The *Runx2*-positive precursors then express *Sp7* to become *Runx2*-*Sp7* double-positive precursors. Runx2 and Sp7 are master regulators of osteoblast development, as osteoblasts are absent in mutant mice lacking either of them [6,7]. The double positive precursors eventually differentiate into osteoblasts and mature, secreting bone matrices. Alkaline phosphatase (*Alpl*), secreted phosphoprotein 1 (*Spp1*, also known as osteopontin), and bone gla protein (*Bglap*, also known as osteocalcin) characterize osteoblast precursors, osteoblasts, and mature osteoblasts, respectively.

**Figure 1:**
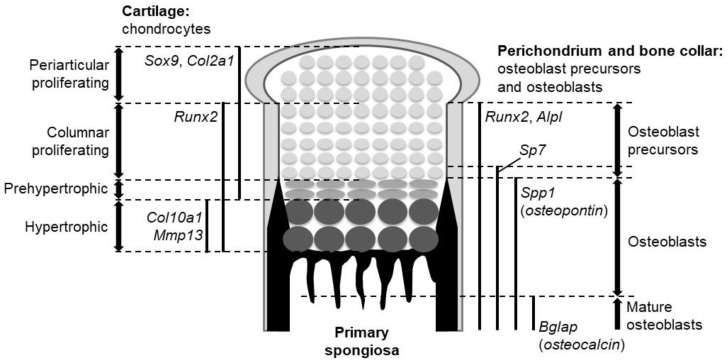
The expression domains of marker genes and the location of the different cell types in the growth plate of endochondral bones.

The fetal cartilage of endochondral skeletons is referred to as the growth plate. In the growth plate, chondrocytes are sequentially layered and aligned according to their differentiation stages: The proliferating stage, prehypertrophic stage, and hypertrophic stage (Figure 1). Importantly, the bone collar is formed at the perichondrium in synchronization with chondrocyte hypertrophy; it always occurs at the perichondrial region adjacent to hypertrophic chondrocytes (Figure 1). These features represent the chronological process of chondrocyte maturation and osteoblast differentiation from the epiphysis toward the diaphysis (Figure 1).

Among the three Hh ligands in mammals, Ihh is notable for its role in endochondral ossification, where it acts as a major Hh input for the biological action of Hh. *Ihh* is expressed strongly in prehypertrophic chondrocytes and weakly in hypertrophic chondrocytes of the growth plate. The expression of *Ptch1* and *Gli1*, readouts of Hh signaling activation, is observed in proliferating chondrocytes, perichondrial cells, and primary spongiosa. Cells closer to the Ihh-producing cells express the readouts at higher levels.

## 3. Hh Signaling Transduction

Hh proteins are synthesized as <45 kDa precursor proteins in Hh-producing cells. The precursor protein is cleaved and divided into two fragments: A 19-kDa N-terminal peptide (Hh-N) and a 25-kDa C-terminal peptide (Hh-C). The Hh-N, which represents all of the Hh signaling activity, is subjected to sequentially occurring dual lipidation: Cholesterol modification to its C-terminus via covalent bonds and a subsequent palmitoylation at its N-terminus. The palmitoylation is mediated by skinny hedgehog (Ski), a membrane-bound O-acyltransferase (MBOAT). The processing and lipidation regulate not only the biological activity of Hh-N, but also affinity for the cell membrane. The affinity controls the range of Hh-N mobility over the tissue and resulting signaling gradient. Hh-C is further digested by the proteasome. The processed Hh-N is transported to the cell surface and released from the Hh-producing cells. Dispatched (Disp), a 12-pass transmembrane protein, regulates the releasing process; *Disp1* and *Disp2* have been identified in mice [1,2,8].

**Figure 2:**
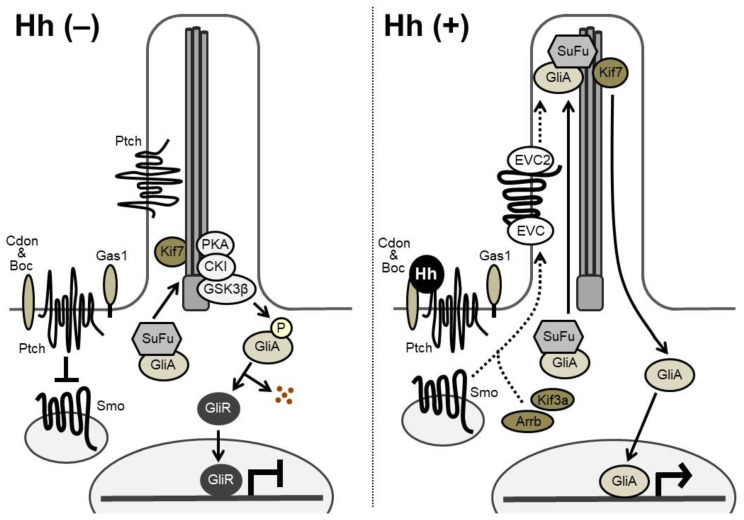
Hh signaling transduction. (**Left**) In the absence of Hh ligands, Ptch inhibits intrinsic Smo activities. The full-length Gli factor (Gli activators: GliA), exists in complex with Suppressor of Fused (SuFu). The full-length GliA is phosphorylated by PKA, CKI, and GSK3β at the base of the cilia. The phosphorylation causes proteolytic cleavage of GliA to generate the transcriptional repressor form of Gli (Gli repressors: GliR). GliR represses transcription of target genes in the nucleus, since it possesses the transcriptional repressor domain and the DNA-binding domain, but lacks the transactivation domain. (**Right**) Binding of Hh ligands relieves the repressive effect of Ptch on Smo. Smo is phosphorylated by G protein-coupled receptor kinase 2 (Gpcrk2) and CKI. Smo also interacts with Kif3a and β-arrestin (Arrb) and accumulates in the cilia in association with Ellis-van Creveld syndrome protein (EVC) and EVC2 (dashed arrows). The levels of GliA and SuFu are increased in the cilia, leading to the dissociation of the GliA-SuFu complex in the cilia. GliA then escapes from proteolytic cleavage and activates transcription of target genes in the nucleus.

Hh signaling transduction is illustrated in Figure 2. In Hh-receiving cells, Smoothened (Smo), a seven-pass transmembrane protein, has an intrinsic intracellular signaling activity that is repressed by Patched (Ptch), a 12-pass transmembrane receptor of Hh. Two *Ptch* genes, *Ptch1* and *Ptch2*, have been identified in vertebrates. Cdon (cell adhesion molecule-related/downregulated by oncogenes), Boc (brother of Cdo), and Gas1 (growth arrest specific 1) are known to act as co-receptors of Ptch, enhancing the binding affinity between Hh and Ptch. Without Hh ligands, Ptch proteins are located mainly around the primary cilia and inhibit the Smo activity. When Hh ligands bind to Ptch on the target cell, Ptch exits from the cilia; the action relieves the repressive effect of Ptch on Smo, which then shuttles through the cilia, initiating Hh signal transduction [1,2,8].

In vertebrates, Hh-responsive transcription is mediated through the zinc finger transcription factors Gli1, Gli2, and Gli3 [1]. Gli1 is one of the target genes of Hh signaling, and functions as a strong transcriptional activator. Gli2 and Gli3 are thought to act as both full-length activator forms and truncated repressor forms. Suppressor of Fused (SuFu) forms a complex with Gli factors and sequesters them in the cytoplasm [2,9]. Without Hh input, Gli2 and Gli3 are phosphorylated by protein kinase A (PKA), casein kinase 1α (CKI), and glycogen synthase kinase 3β (GSK3β) and processed into transcriptional repressor forms at the base of the cilia [2]. Speckle-type POZ protein (Spop), a substrate-binding adaptor for the cullin3-based ubiquitin E3 ligase, targets Gli2 and Gli3 for ubiquitination and proteasomal degradation [2,9,10]. Upon Hh input, released Smo prevents proteolytic processing of Gli2 and Gli3. Thus, the Gli-activator forms translocate into the nucleus to activate the transcription of target genes, while a decrease in Gli transcriptional repressors induces derepression of another set of genes. Gli2 was suggested to function primarily as a transcriptional activator, and Gli3 as a transcriptional repressor, although a few studies have shown the opposite [1].

## 4. Roles of Hh Signaling in Endochondral Ossification

**Figure 3:**
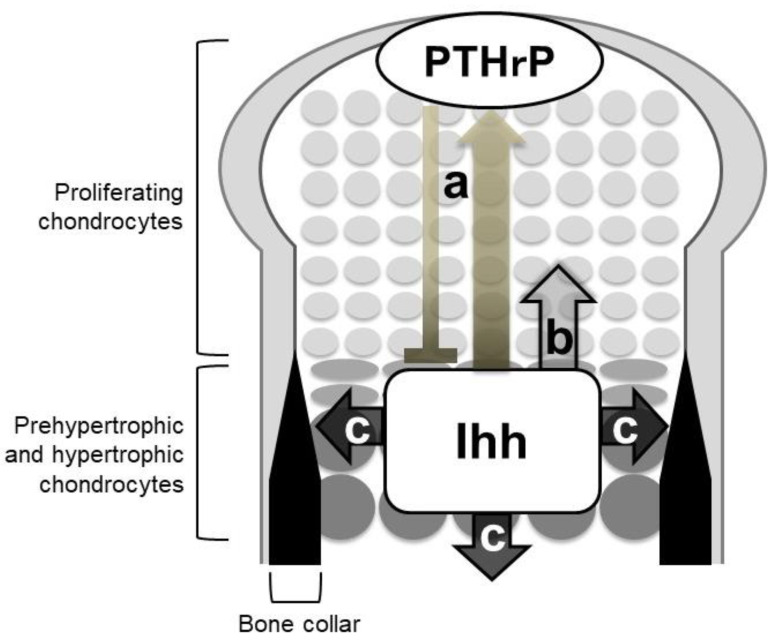
Roles of Indian hedgehog (Ihh) in endochondral ossification. Mouse genetic studies support three major roles of Ihh in endochondral ossification: (**a**) Chondrocyte differentiation via the negative feedback loop with parathyroid hormone-related protein (PTHrP), (**b**) chondrocyte proliferation, and (**c**) specification of bone-forming osteoblasts. Ihh acts on chondrocyte proliferation and osteoblast specification in a direct manner.

The phenotypes of *Ihh*-null mutant mice clearly demonstrated the three Hh signaling-dependent processes in endochondral ossification (Figure 3): (1) Chondrocyte differentiation, (2) chondrocyte proliferation, and (3) specification of bone-forming osteoblasts. Other genetic studies have supported the role of Hh signaling with some mechanistic insights. Ihh indirectly regulates the chondrocyte differentiation via a negative feedback loop with parathyroid hormone-related protein (PTHrP), whereas it acts on chondrocyte proliferation and osteoblast specification in a direct manner.

### 4.1. An Ihh-PTHrP Negative Feedback Loop Maintains the Growth Plate Length

Periarticular proliferating chondrocytes and the perichondrium express PTHrP (officially named as parathyroid hormone like hormone: PTHLH), whereas parathyroid hormone 1 receptor (*PTH1R*; also described as the PTH/PTHrP receptor or PPR) is expressed strongly in prehypertrophic chondrocytes and weakly in proliferating chondrocytes [11,12]. The phenotypes of mutant mice with the deletion of *Pthrp*, *Ppr*, or both *Pth* and *Pthrp* [13,14,15,16] and those with the chondrocyte-specific overexpression of *Pthrp* or the constitutively active *Ppr* [17,18,19] suggested that PTHrP suppressed chondrocyte hypertrophy via PPR and thereby kept chondrocytes proliferating.

The role of Ihh in association with the action of PTHrP was initially proposed by Lanske et al. and Vortkamp et al. in 1996 [15,20]. Vortkamp et al. demonstrated that *Ihh* overexpression in chick limbs suppressed chondrocyte hypertrophy with increased *Pthrp* expression in the periarticular perichondrium [20]. The addition of PTHrP rescued premature hypertrophy of *Pthrp*^−/−^ mouse limbs, whereas Shh had no effect on *Pthrp*^−/−^ mouse limbs. This result suggests that Hh acts upstream of PTHrP in the growth plate. In the work by Lanske et al., PTHrP and Shh treatment both induced elongation of the growth plate with the suppression of chondrocyte hypertrophy in wild-type limbs, although neither of them had effects on *Ppr*^−/−^ limbs [15]. These two studies indicated that the transition from proliferating chondrocytes to hypertrophic ones was regulated by both PTHrP-PPR signaling and Ihh; they constitute a common feedback loop, where the PTHrP-PPR signaling mediated the effect of Ihh on the transition.

In 1999, St-Jacques et al. reported skeletal phenotypes of *Ihh*^−/−^ mice [21]. *Ihh*^−/−^ mice showed acceleration of chondrocyte hypertrophy, which resulted in shortening of the proliferating chondrocyte layer, with loss of *Pthrp* expression in the periarticular regions [21]. Thus, the *Ihh*^−/−^ mice phenocopied the mice with the loss of PTHrP function in terms of the growth plate structure. In contrast, activation of Hh signaling in chondrocytes by the loss of *Ptch1* caused a delay of chondrocyte hypertrophy and upregulation of *Pthrp* expression in mice [22]. These studies supported the aforementioned link between Ihh and PTHrP.

Karp et al. genetically determined the link by analyzing the phenotypes of compound mutant mice [23]. *Ihh*^−/−^;  *Pthrp*^−/−^ mutant limbs exhibited abnormalities similar to those of *Ihh*^−/−^ mutant limbs [23]: Premature hypertrophy of chondrocytes and lack of the growth plate, trabecular bone, and bone collar. Importantly, activation of PTHrP signaling in the *Ihh*^−/−^ mutant background cancelled the premature hypertrophy of *Ihh*^−/−^ chondrocytes, but did not increase the number of mitotically active chondrocytes [23]. These results suggest that: (1) Ihh is required for both the differentiation and the proliferation of growth plate chondrocytes; (2) PTHrP partly mediates the Ihh function in maintaining a pool of proliferating chondrocytes; and (3) Ihh positively regulates chondrocyte proliferation in a PTHrP-independent manner.

To gain insight into the specific cellular interaction underlying the functional Ihh-PTHrP link, Chung et al. analyzed the growth plate of chimeric mice carrying *Ppr*^−/−^ cells and WT cells [24]. *Ppr*^−/−^ chondrocytes ectopically became hypertrophic in columnar proliferating regions and expressed *Ihh*. The chimeric mice also showed the upregulation of *Pthrp* in periarticular WT cells and an elongation of the growth plate. In chimeric mice carrying *Ppr*^−/−^; *Ihh*^−/−^ cells and WT cells, *Ppr*^−/−^; *Ihh*^−/−^ chondrocytes still exhibited ectopic hypertrophy, but the upregulation of *Pthrp* and the elongation of the growth plate were no longer observed [25].

**Figure 4:**
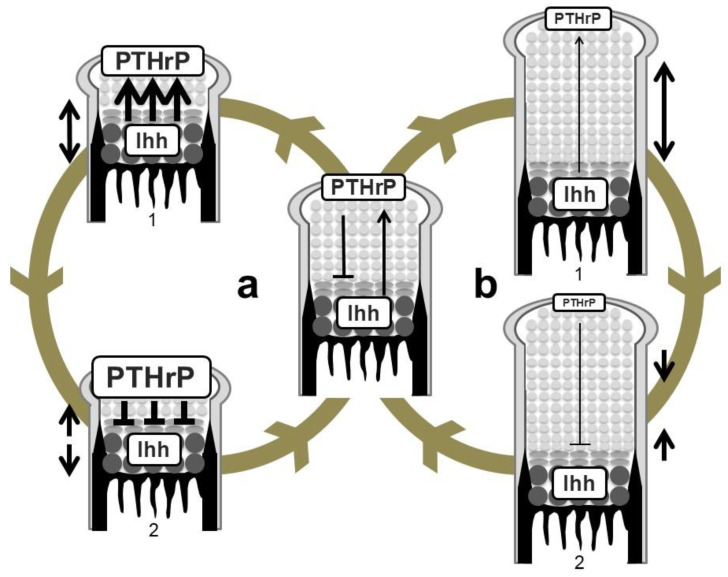
Regulation of growth-plate length by the Ihh-PTHrP negative feedback loop. (**a**) Shortening of the length of the growth plate (double-headed arrow on the left of 1) increases PTHrP expression in periarticular proliferating chondrocytes possibly due to the increase in the amount of Ihh reaching the PTHrP-producing population (wide arrows in 1). This causes greater suppression of hypertrophy (wide lines in 2) compared to an equilibrium state. The growth plate thereby elongates to restore the equilibrium state (head-to-head arrows on the left of 2). (**b**) Elongation of the growth plate (double-headed arrow on the right of 1) in turn decreases PTHrP expression due to the decrease in the amount of Ihh reaching the PTHrP-producing population (thin arrow in 1). PTHrP activity on hypertrophy is attenuated (thin line in 2), leading to shortening of the length of the growth plate (head-to-head arrows on the right of 2).

The above-described genetic studies establish the idea of an Ihh-PTHrP negative feedback loop. Ihh and PTHrP both contribute to maintenance of the growth plate length via the following functional loop (Figure 3 and Figure 4): Ihh produced by prehypertrophic chondrocytes facilitates the expression of PTHrP in cells around the periarticular regions, i.e., periarticular chondrocytes and perichondrial cells. Ihh possibly regulates the PTHrP expression in a concentration-dependent manner by acting as a morphogen. PTHrP then exerts a negative impact on chondrocyte hypertrophy by acting on PPR-expressing prehypertrophic chondrocytes. The suppressive effect of PTHrP on hypertrophy keeps chondrocytes proliferating, which leads to an increase in the distance between PTHrP- and Ihh-producing cells. This functional loop maintains a pool of proliferating chondrocytes by tightly regulating the distance; changes in the distance alter the PTHrP expression level at the periarticular region, leading to the correction of the distance (Figure 4). For example, reduction of the distance between PTHrP- and Ihh-producing cells facilitates PTHrP expression, probably due to the increased amount of Ihh acting on the periarticular region, and thereby the distance is increased, and vice versa. This notion is supported by the growth plate phenotype of *Ppr*^−/−^/WT chimeric mice, in which ectopic hypertrophy of *Ppr*^−/−^ chondrocytes occurred much closer to the periarticular region than that of WT chondrocytes, and the length of the columns of WT proliferating chondrocytes was increased with enhanced *Pthrp* expression at the periarticular region. Thus, the Ihh-PTHrP negative feedback loop maintains a certain length of the growth plate in order to maximize skeletal growth.

Several studies support the requirement of direct Ihh input for *Pthrp* expression in periarticular proliferating chondrocytes and the periarticular perichondrium. When *Smo* was removed specifically from subsets of growth plate chondrocytes in mice, *Pthrp* expression became little or absent in the periarticular domain where Hh signaling was not activated [26]. Removal of the perichondrium from chicken embryonic tibiotarsi caused the expansion of the hypertrophic chondrocyte layer, and the addition of parathyroid hormone rescued the expansion [27]. As mentioned earlier, Ihh overexpression increased *Pthrp* expression in the periarticular perichondrium [20]. The latter two studies specifically suggest the involvement of the periarticular perichondrium as a source of PTHrP in the Ihh-PTHrP negative feedback loop.

### 4.2. Ihh Directly Regulates the Proliferation of Growth Plate Chondrocytes

The decreased proliferation of chondrocytes in *Ihh*^−/−^ mice and chondrocyte-specific *Smo*^−/−^ mice [21,28] indicates the direct and positive regulation of chondrocyte propagation by Ihh input in the growth plate (Figure 3), as Karp et al. suggested [23]. Hh signaling has also been shown to directly regulate the differentiation of growth plate chondrocytes at multiple steps independently of PTHrP. Kobayashi et al. proposed that Ihh drove the differentiation of periarticular proliferating chondrocytes into columnar proliferating chondrocytes in a PTHrP-independent manner [29,30]. In addition, chondrocyte-specific removal of *Smo* delayed chondrocyte hypertrophy without PTHrP in mice [31].

### 4.3. Ihh Is Required for Specification of Osteoblasts

Hh signaling act as a master regulator of osteoblast development during endochondral ossification. In endochondral bones, osteoblasts first emerge in a perichondrial region adjacent to pre-hypertrophic and hypertrophic chondrocytes. Mouse genetic studies demonstrate that Ihh produced by these chondrocytes acts on osteoblast progenitors to execute an osteoblast specification program in the perichondrium and the primary spongiosa. *Ihh*^−/−^ mice showed no bone collar and lacked *Runx2* and *Bglap* expressions in the perichondrium [21,32]. The analysis of *Ppr*^−/−^/WT chimeric mice and *Ppr*^−/−^; *Ihh*^−/−^/WT chimeric mice further supported the involvement of Ihh and Ihh-producing cells, i.e., pre- and hypertrophic chondrocytes, in bone collar formation [24,25]. In *Ppr*^−/−^/WT chimeras, ectopic calcification was observed in the perichondrium adjacent to ectopically hypertrophic *Ppr*^−/−^ cells. The ectopic calcification disappeared in *Ppr*^−/−^; *Ihh*^−/−^/WT chimeras, although *Ppr*^−/−^; *Ihh*^−/−^ cells still showed ectopic hypertrophy with robust expressions of bone morphogenetic protein (*Bmp*) *2* and *6*. These chimeric mouse studies suggest that hypertrophic chondrocytes induce bone formation at their adjacent regions by secreting Ihh. Another important point is that BMP2 and 6 produced by hypertrophic chondrocytes may not be sufficient to induce bones at this step.

The requirement of direct Hh input for osteoblast specification is supported by the phenotypes of *Smo* mutant mice. Ablation of *Smo* from perichondrial cells caused absence of *Runx2* expression and bone collar formation in the perichondrium [33]. In *Smo*^−/−^/WT chimeric mice, *Smo*^−/−^ perichondrial cells, which did not differentiate into osteoblasts, expressed chondrocyte marker genes including type II collagen (*Col2a1*) and type X collagen (*Col10a1*) in the region where the bone collar is formed under physiological conditions. *Smo^−/−^* cells did not contribute to bone-forming regions in the primary spongiosa [33]. In contrast, the chondrocyte-specific overexpression of *Ihh* and the ablation of *Ptch1* from perichondrial cells led to acceleration of bone collar formation, accompanied by the activation of Hh signaling in the perichondrium [22,33].

The above-described studies demonstrate that Ihh produced by pre- and hypertrophic chondrocytes is essential for osteoblastogenesis in the perichondrium and the primary spongiosa during endochondral ossification (Figure 3). In particular, Hh signaling is necessary for the specification of progenitors into *Runx2*-positive osteoblast precursors. As *Smo* deletion in *Sp7*-positive cells showed no obvious abnormality in osteoblast development, Hh signaling is dispensable for the later phase of osteoblastogenesis [34]. Given that chondrocytic phenotypes were acquired by perichondrial cells that cannot receive Hh input [33], Hh signaling may specify the cell fates of the osteo-chondroprogenitor population into the osteoblastic lineages in the perichondrium.

### 4.4. Factors Acting Downstream of Hh Signaling During Endochondral Ossification

Which Gli factors act downstream of Ihh in endochondral ossification? In regulation of growth plate chondrocytes, Ihh is likely to utilize Gli3 to exert its biological effects. Removal of *Gli3* on an *Ihh^−/−^* background rescued abnormalities in the proliferation and maturation of chondrocytes in *Ihh^−/−^* mice [32,35]. The *Gli3*^−/−^; *Ihh*^−/−^ mice also showed recovery of *Pthrp* expression in the periarticular regions [32,35]. Together with the fact that Gli3 primarily acts as a transcriptional repressor, Ihh-mediated suppression of the Gli3 repressor activity may derepress the *Pthrp* expression in an Ihh-PTHrP negative feedback loop. Because neither *Gli1*^−/−^, *Gli2*^−/−^*,* nor *Gli1*^−/−^; *Gli2*^−/−^ mouse embryos demonstrated any obvious defects in the growth plate [16,36,37,38], Gli1 and Gli2 are unlikely to have dominant roles in maintenance of the growth plate.

In contrast, osteoblast development is mediated by all of the Gli factors, Gli1, Gli2, and Gli3, upon Hh input. The activator function of Gli2 was initially a focus in this context. Shimoyama et al. reported that Ihh induced osteoblast differentiation in a Gli2-dependent manner; they proposed that Runx2 induction and the physical interaction between Runx2 and Gli2 underlay the Ihh-Gli2 axis [39]. Joeng and Long provided further evidence supporting the importance of Gli2 in osteoblastogenesis; the skeletal phenotypes of *Ihh^−^*^/*−*^ embryos were completely rescued in *Ihh^−^*^/*−*^; *Gli3^−^*^/*−*^; *C2*-*NGli2* embryos, in which *NGli2* (an N-terminally truncated, constitutively active form of Gli2) was exogenously expressed in *Col2a1*-positive cells under an *Ihh^−^*^/*−*^; *Gli3*^−/−^ background. Based on this result, they proposed that the Gli2 activator and the Gli3 repressor collectively mediated all major aspects of the Ihh function in endochondral ossification [40].

We have proposed that Gli1 is also involved in Hh-mediated osteoblast specification cooperatively with Gli2 and Gli3, based on the following findings: First, *Gli1*^−/−^ mice showed impairment of bone formation [37]. Second, *Gli1*^−/−^ perichondrial cells expressed *Col2a1* and *Col10a1*, but not *Runx2* or *Sp7* [37]. Third, *Gli1*^−/−^;*Gli2*^−/−^ mice showed more severe skeletal phenotypes than either *Gli1*^−/−^ or *Gli2*^−/−^ mice [37]. Fourth, osteoblast differentiation was impaired in *Gli1*^−/−^; *Gli3*^−/−^ perichondrial cells compared to that in *Gli3*^−/−^ cells in vitro [37]. Lastly, Gli1 activated the transcription of early and middle marker genes for osteoblasts by directly binding to the 5′ regulatory regions of the genes [37], and it interfered with the Sox9-mediated transactivation of chondrocyte marker genes by suppressing the DNA binding of Sox9 [38]. We also found that the Gli3 repressor suppressed the Runx2-mediated transcription of osteoblastic genes by antagonizing the DNA binding of Runx2 [41].

Given that *Runx2* expression is lost in the perichondrium of mice defective with Hh signaling, one might expect that Runx2 is involved in Hh-mediated osteoblast specification. However, the recovery of *Runx2* expression in the *Ihh*^−/−^ perichondrium did not cancel the abnormality of osteoblast differentiation in *Ihh*^−/−^ mice [42]. Thus, Runx2 alone is unlikely to account for the function of Hh signaling in this context.

## 5. Roles of Hh Signaling in Craniofacial Development

Key features that distinguish cranial skeletal components forming the face and the rostral cranial vault from others in the head, limbs, and trunk are that the former components (1) originate from the cranial neural crest cells (CNCCs) and (2) generate osteoblasts via the intramembranous ossification.

Neural crest cells are multipotent and self-renewing. Notably, they have a greater capacity for differentiation than the cells from which they originate: They give rise to not only ectodermal lineages, but also mesodermal lineages. In vertebrates, the neural crest progenitors are derived from the ectodermal germ layer, arising in the neural plate border, a region between the neural plate and non-neural ectoderm that forms the future epidermis. The induction of the neural plate border, i.e., the induction of neural crest progenitors, is mediated by a dynamic interplay of patterning signaling pathways: BMP, FGF, Wnt, and Notch (extensively reviewed in [43]).

Hh signaling plays indispensable roles in the formation of CNCC-derived cranial skeletons by acting on postmigratory CNCCs. Jeong et al. found that the removal of *Smo* in CNCCs using the *Wnt1-Cre* driver resulted in extensive loss of CNCC-derived skeletal elements in mice, although the generation and migration of CNCCs were not affected and the mesoderm-derived skeletons remained intact [44]. They further found that five Forkhead box (*Fox*) family transcription factors (*Foxc2*, *Foxd1*, *Foxd2*, *Foxf1*, and *Foxf2*), which were selected through a transcriptional profiling of *Shh* mutant and WT heads, were specifically downregulated in craniofacial regions of the CNCC-specific *Smo* mutants, which suggested that the *Fox* genes were direct and major mediators of the function of Hh in craniofacial development [44]. Importantly, *Smo* deletion affected not only intramembranous bones, but also endochondral bones including rostral half of the basisphenoid. Thus, the involvement of Fox genes in craniofacial development may be supported by a recent paper, which demonstrates that Fox genes are necessary for Sox9-mediated induction of chondrogenic genes in zebrafish [45].

Shh is thought to be responsible for this action, since *Shh*, but not *Ihh,* is expressed in epithelial populations in the developing face from E9.5 to E12.5; *Ptch1* is expressed not only in the epithelium, but also in the mesenchyme, which contains a high density of CNCCs [44], and this fact supports the functional importance of the signaling in this population. Although Ihh is unlikely to play major roles in this context, *Ihh* mutants have mild craniofacial defects [21].

The expressions of Shh and Ihh in murine cranial sutures have been debated (reviewed in [8]). Ihh has been shown to be expressed in ossifying bones and osteogenic fronts, whereas Shh is expressed in the suture mesenchyme [8,46]. Based on findings in *Ihh*^−/−^ mouse cranial skeletons and the *Ihh* misexpression in chick embryonic heads, Abzhanov et al. speculated that in cranial intramembranous bones, the *Ihh* produced by mature osteoblasts acts as a feedback-inhibitor for the transition of pre-osteoblasts to the chondrocyte-like osteoblasts, which they proposed, and mature osteoblasts [47]. Amano et al. recently reported phenotypes of mutant mice in which *Ihh* was deleted in CNCCs by a *Wnt1-Cre* driver line [48]. The mutant showed disruption of the midface structure, where intersphenoid synchondrosis and nasal cartilage were greatly impaired, and a shortened mandible. This study also supports the idea that Ihh plays roles in the craniofacial skeleton.

Mutations in cilium-related factors caused abnormalities in cranial skeletons, in line with the fact that Hh signaling transduction takes place at the primary cilia. In particular, mutations of several intraflagellar transport proteins (IFTs), which are involved in Hh signaling transduction at primary cilia, have been implicated in craniofacial development. These IFTs include IFT144 [49] and Kif3a [50,51].

## 6. Postnatal Roles of Hh in Skeleton

Several genetic studies have highlighted the roles of Hh signaling in postnatal cartilage and the growth plate. When *Ihh* was deleted from *Col2a1*-expressing cells at the postnatal stages in mice, ectopic chondrocyte hypertrophy, decreased proliferation of chondrocytes, and a disorganized growth plate was observed [52]. Postnatal deletion of *Ihh* in *Prrx1*-expressing skeletal progenitors caused lack of the growth plate and a secondary ossification center in mice [53]. Thus, Ihh is necessary for postnatal maintenance of the growth plate and progression of ossification in endochondral bones. Ihh can function in both PTHrP-dependent and -independent manners in this context; the forced expression of constitutively active *Ppr* temporally cancelled the ectopic chondrocyte hypertrophy led by the deletion of *Ihh* from *Col2a1*-expressing cells, but did not correct the decreased proliferation of chondrocytes [54]. These data suggest the following. (1) Ihh and PTHrP functionally interact to control hypertrophy of growth plate chondrocytes at postnatal stages, as they do at embryonic stages. (2) Ihh promotes chondrocyte proliferation at the postnatal stage independently of PTHrP-PPR signaling. In line with the first notion, chondrocyte-specific ablation of *Ppr* in postnatal mice led to the acceleration of hypertrophy, followed by premature closure of the growth plate, in association with increased chondrocyte apoptosis [55]. However, the work by Maeda et al. also suggests that the contribution of the Ihh-PTHrP interaction to chondrocyte hypertrophy may occur less at postnatal stages than at embryonic stages [52].

We and others have demonstrated that Hh signaling also regulates postnatal bone mass. *Ptch1*^+/−^ mice showed high bone mass with a high bone turnover phenotype in adults; both bone formation and bone resorption were accelerated in the mutant mice [41]. The mouse phenotype was partly recapitulated in patients with Gorlin syndrome caused by inactivating mutations of one of the *PTCH1* alleles [41]. *Ptch1* deletion in *Bglap*-expressing mature osteoblasts also led to high bone turnover in mice [56]. However, unlike *Ptch1*^+/−^ mice, the mutant mice showed fragile long bones with low bone mass [3,56]. These results indicate that indirect effects of Hh signaling on osteoclasts may affect bone metabolism more than its direct effects on osteoblasts, when Hh signaling was activated specifically in mature osteoblasts. Indeed, the expression of Receptor activator of nuclear factor kappa-B ligand (RANKL) was increased in osteoblasts of both mutants. Mak et al. further showed the involvement of the PTHrP-PKA-CREB (cAMP responsive element binding protein) axis in upregulation of the RANKL expression upon Hh signaling activation in mature osteoblasts [56]. We also reported the effect of attenuation of Hh signaling on adult bone mass; *Gli1* haploinsufficiency resulted in decreased bone mass with reduced bone formation and accelerated bone resorption, and impairment of fracture healing in adult mice [57]. The osteoblast phenotype of *Gli1*^+/−^ mice supports the positive roles of Hh signaling in osteoblast differentiation. However, it is still debated how Hh signaling regulates osteoclastogenesis. Studies so far suggest not only its indirect actions via osteoblasts, but also direct actions on osteoclast precursors [58], and the signaling is likely to exert both positive and negative effects on osteoclastogenesis in context- or stage-dependent manners. In addition, regarding the involvement of Hh signaling in human adult bones, we need to consider that the growth plate, a major source of Ihh in the skeleton, is closed after puberty in humans. The alternative source of Hh ligands and its contribution to adult bone metabolism remain to be fully elucidated.

The postnatal expression of Ihh in the articular cartilage has been implicated in osteoarthritis (OA). Ihh expression is increased in human OA cartilages, whereas the expression is at the low level in healthy cartilages [59]. Postnatal deletion of *Ihh* attenuated OA progression in a surgically induced OA model compared to the WT mice [60]. Activation of Hh signaling in turn developed OA in mice [61]. Thus, Ihh can be a therapeutic target for the treatment of OA; suppression of the Ihh activity or its downstream signaling in the articular cartilage may modify OA progression or even inhibit its development in adults.

Recently, postnatally existing skeletal stem cell-like populations have drawn attention [62,63,64]. Shi et al. reported that Gli-positive cells worked as metaphyseal mesenchymal progenitors (MMPs) at the postnatal stage [65]. The Gli1-positive MMPs, residing immediately below the growth plate, expressed a set of mesenchymal stem cell markers and produced cancellous bone osteoblasts in postnatal mice. They also found that the Gli1-positive MMPs contribute to fracture healing by giving rise to both osteoblasts and chondrocytes. Haraguchi et al. also investigated the cell fate of *Gli1*-expressing cells present in hypertrophic chondrocytes and the chondro-osseous junction at postnatal stages in mice [66]. In this context, *Gli1*-expressing cells are supposed to be Ihh-responding cells in and around the growth plate. Descendants of the *Gli1*-expressing cells contributed to osteoblastic cells in the periosteum, trabecular bones, and cortical bones [66]. These data suggest that Hh-responding cells are involved in physiological bone formation at postnatal stages as well as embryonic ones.

Lastly, we recently demonstrated the positive role of Hh signaling in human osteoblastogenesis and its therapeutic potential by utilizing disease-specific induced pluripotent stem cells (iPSCs) [67]. We focused on two genetic diseases, Gorlin syndrome (see also the next Section 7.) and McCune Albright syndrome (MAS). Gorlin syndrome-derived iPSCs showed augmentation of osteoblastogenesis with Hh signaling activation, whereas MAS-specific iPSCs showed impaired osteoblastogenesis with low Hh signaling activity. Importantly, impaired osteoblastogenesis of MAS-specific iPSCs was restored by treatment with a Smo agonist.

## 7. Skeletal Diseases Caused by Abnormalities of Hh Signaling

Consistent with its critical roles revealed by mouse genetic studies, disruption of normal Hh signaling is known to cause human skeletal diseases. GWAS for genetic variants influencing human height identified *IHH* and Hh signaling components, *PTCH1* and *HHIP* (hedgehog interacting protein), as height-associated loci [68,69]. This result supports the prerequisite role of Ihh in maintenance of the growth plate via the negative feedback loop formed together with PTHrP, in order to maximize skeletal growth.

Most of other Hh-related skeletal diseases are characterized by patterning defects. Heterozygous mutations of *IHH* also cause brachydactyly type AI (BDA1; OMIM number: 112500), in which the middle phalanges of all the digits are rudimentary or fused with the terminal phalanges [70,71,72]. Mice carrying one of the BDA1 mutations, E95K, recapitulated the phenotype of human patients with BDA1 [72]. Phalanges of the heterozygous BDA1 mutant mice were mildly affected, whereas homozygous mutants showed a classic BDA1 phenotype [72]. Altered expression pattern of *Ptch1*, a readout of Hh signaling, in the mutants’ growth plate indicates a change in the Ihh activity gradient, probably due to decreased binding capacity of the E95K-Ihh mutant to the receptor Ptch1 [72]. Greig cephalopolysyndactyly syndrome (GCPS; OMIM number: 175700), Pallister-Hall syndrome (PHS; OMIM number: 146510), and postaxial polydactyly type A1 and type B3 (PAPA1 and PAPB; OMIM number: 174200) are resulted from heterozygous mutations of *GLI3* [73,74,75]. These diseases are characterized by various bone-related anomalies, including abnormalities of skull or limbs, syndactyly, and postaxial polydactyly. Gorlin syndrome, also known as basal cell nevus syndrome (BCNS; OMIM number: 109400), is a rare autosomal-dominant disorder caused by heterozygous inactivating mutations of *PTCH1*. Patients with Gorlin syndrome show skeletal abnormalities, craniofacial abnormalities, large body size, and tumors, including basal cell carcinomas of the skin and cerebellar medulloblastomas [76].

Impaired Hh signaling is thought to underlie Smith-Lemli-Opitz syndrome (SLOS; OMIM number: 270400), an autosomal recessive multiple congenital malformation and mental retardation syndrome. SLOS is caused by mutations of the gene encoding the cholesterol biosynthetic enzyme 7-dehydrocholesterol reductase (DHCR7) [77], and its clinical features include polydactyly and syndactyly as seen in the aforementioned diseases with aberrant Hh signaling. Indeed, the *DHCR7* mutations perturb its ability to catalyze the conversion of 7-dehydrocholesterol (7DHC) to cholesterol; the reduced cholesterol level affects Hh signaling due to reduced Smo activities [78].

Aberrant Hh signaling was recently associated with ectopic ossification. Progressive osseous heteroplasia (POH; OMIM number: 166350) is known to show progressive ankylosis and growth retardation caused by ectopic ossification due to mutations in *GNAS*, which encodes Gαs [79]. Regard et al. found that patients with POH showed upregulation of Hh signaling; they also demonstrated that GNAS inhibited Hh signaling through cAMP-mediated PKA activation, and suppression of the Hh signaling activity partially rescued the POH phenotypes [80]. Thus, Hh signaling activation by *GNAS* mutation is likely to underlie heterotopic ossification in POH.

## 8. Conclusions

As reviewed in this paper, understanding of the Hh signaling function in skeletal development has greatly advanced in the last two decades. Hh signaling not only couples chondrogenesis with osteogenesis during endochondral ossification, but also regulates intramembranous ossification in the cranial skeleton. In addition, the molecular mechanisms underlying the function have been revealed with particular focuses on Gli transcription factors and other signaling components. However, two central questions remain to be addressed. First, the gene regulatory networks underlying these actions are unclear. What genes act downstream of the Hh-Gli axis in the skeleton? What transcription factors engage the Gli-mediated network during skeletal development? The recent advancement in next generation sequencer (NGS)-based genome-wide analyses and single-cell analysis will provide insight into these questions. Second, the regulatory landscape underlying *Ihh* transcription is still poorly understood. How do the enhancer-promoter interaction and distal enhancer networks regulate the *Ihh* transcription? Is the disruption of the network relevant to human disease? Recently, Will et al. identified five enhancer regions upstream of the *Ihh* gene; they have overlapping activities in vivo, and a cluster of the redundant enhancers regulates the *Ihh* expression in skeletal development [81]. The core enhancer elements and transcription factors engaging the elements remain to be identified. Thus, further studies are expected to elucidate the gene regulatory networks of *Ihh* transcription itself and those downstream of the Hh-Gli axis. This knowledge will enable us to understand the fate specification and subsequent maturation of skeletal cells, and the potential link between disruption of the process with human diseases when combined with GWAS and/or disease-specific iPSCs.

## Abbreviations

Hh	Hedgehog
Ihh	Indian hedgehog
PTHrP	parathyroid hormone-related protein
Shh	Sonic hedgehog
Dhh	Desert hedgehog
MMP13	matrix metalloproteinase 13
VEGF	vascular endothelial growth factor
Runx2	runt-related transcription factor 2
Alpl	alkaline phosphatase
Spp1	secreted phosphoprotein 1
Bglap	bone gla protein
Ski	skinny hedgehog
MBOAT	membrane-bound O-acyltransferase
Disp	Dispatched
Smo	Smoothened
Ptch	Patched
Cdon	cell adhesion molecule-related/downregulated by oncogenes
Boc	brother of Cdo
Gas1	growth arrest specific 1
SuFu	Suppressor of Fused
PKA	protein kinase A
CKI	casein kinase 1α
GSK3β	glycogen synthase kinase 3β
Spop	Speckle-type POZ protein
Gpcrk2	G protein-coupled receptor kinase 2
Arrb	β-arrestin
EVC	Ellis-van Creveld syndrome protein
PTH1R	parathyroid hormone 1 receptor
PPR	PTH/PTHrP receptor
WT	wild-type
BMP	bone morphogenetic protein
CNCC	cranial neural crest cell
FGF	Fibroblast growth factor
Fox	Forkhead box
IFT	intraflagellar transport proteins
RANKL	Receptor activator of nuclear factor kappa-B ligand
CREB	cAMP responsive element binding protein
OA	osteoarthritis
MMP	metaphyseal mesenchymal progenitor
iPSC	induced pluripotent stem cell
MAS	McCune Albright syndrome
GWAS	genome-wide association study
HHIP	hedgehog interacting protein
BDA1	brachydactyly type AI
GCPS	Greig cephalopolysyndactyly syndrome
PHS	Pallister-Hall syndrome
PAPA1	postaxial polydactyly type A1
PAPB	postaxial polydactyly type B3
BCNS	basal cell nevus syndrome
SLOS	Smith-Lemli-Opitz syndrome
DHCR7	7-dehydrocholesterol reductase
7DHC	7-dehydrocholesterol
POH	progressive osseous heteroplasia
NGS	next-generation sequencers
